# A Simple Model for Assessing Millimeter-Wave Attenuation in Brownout Conditions

**DOI:** 10.3390/s22228889

**Published:** 2022-11-17

**Authors:** Arkadi Zilberman, Natan Kopeika

**Affiliations:** 1 School of Electrical and Computer Engineering, Ben-Gurion University of the Negev, Beer-Sheva 84105, Israel; 2 Gaza Envelope R&D Center, P.O. Box 100, Netivot 8771002, Israel

**Keywords:** mm-wave attenuation, scattering, power transfer, sandstorm, brownout

## Abstract

Flying helicopters in adverse environmental conditions, such as low heights in arid regions, can be dangerous, especially during landing and take-off, since during hovering, the rotors produce a dust cloud of particles. This phenomenon is known as the “brownout” condition. Unlike visible and infrared systems, the radar devices in the microwave or millimeter wave region offer the capability of sufficient transmission through atmospheric obscurants, such as fog, smoke, sand/dust storms, and brownout. In this work, we present a theoretical evaluation of mm-wave (85–100 GHz) attenuation/scattering and power transfer in brownout conditions. The model includes attenuation/scattering prediction and radiant flux, or power collected by the receiver. We are considering the case of sand grain clouds created by helicopter rotor airflow during landing in arid areas. The evaluated scenarios are brownout environments over ranges up to 50 m. The predicted values from the mathematical model are compared with findings in the field and the literature. A simple model for mm-wave power transfer estimation shows satisfactory agreement with the measured values.

## 1. Introduction

### 1.1. Background

Flying helicopters in adverse environmental conditions, such as low heights in arid regions, can be dangerous, especially during landing and take-off, since during hovering, the rotors produce a dust cloud of particles. This phenomenon is known as the “brownout” condition.

To the NATO report [1], a brownout is responsible for approximately 75% of helicopter collision mishaps during operations in arid climates (e.g., Africa, the Middle East, etc.). Moreover, brownouts have remained particularly troublesome for helicopter emergency medical services (HEMS), which frequently need to land in difficult conditions. In addition, a brownout is an example of a Degraded Visual Environment (DVE), a topic that continues to be of high priority in civil and military communities.

Recently, military operations and HEMS in desert environments have brought forth renewed efforts to seek methods for brownout mitigation.

Unlike visible and infrared systems, the radar devices in the microwave or millimeter wave (MMW) region offer the capability of sufficient transmission through atmospheric obscurants, such as fog, smoke, sand/dust storms, and brownout. Thus, the MMW-based solution can be considered an appropriate option for brownout effect mitigation. In addition, atmospheric attenuation for MMW signal propagation is relatively low (~0.1 dB/km) for several frequency bands (e.g., 35, 94, 140 GHz) and low humidity conditions.

Over the past 10–12 years, there have been several DVE solution programs implemented by the DoD and private companies. The US Army’s Helicopter Autonomous Landing System (HALS) has been successfully flight-tested on an EH-60L helicopter [2]. The HALS system relies on a 3D scanning 94 GHz pulsed radar. Another example of a technological solution for brownout mitigation that was successfully tested is the Three-Dimensional Landing Zone (3D-LZ) system [3].

The attenuation problem of MMW signals caused by sand/dust storms has been discussed in the literature for several decades. To date, a relatively moderate number of works exist in the open literature that is tailored to the modeling of MMW propagation and radiative transfer in brownout conditions. Many of the works concentrated on brownout cloud dynamics, formation, and transport [4]. 

Researchers have proposed several models to predict the degree of microwave/MMW signal attenuation caused by dust/sandstorms [5,6,7,8,9,10,11,12,13,14] and sand grain clouds created by helicopter rotor airflow during take-off and landing [15]. Moreover, a theoretical evaluation of a subterahertz (sub-THz) system to image through a scattering medium composed of scatterers of sizes close to the wavelength has been presented [16]. 

In [16], a model has been proposed to determine the performance of an active sub-THz imaging system (above ~95 GHz) in brownout conditions. The authors evaluate the power detected by pixels in the image plane. The main contributions to the power, i.e., the backscattered signal, the backscattered noise, and thermal noise, were determined analytically. The attenuation of the THz wave was estimated by the total extinction mean free path *l*_tot_. The *l*_tot_ is related to the extinction cross-section of a particle of radius *r* and the particle density per unit radius. Estimation of extinction cross section has been proposed using Mie theory.

In this work, we present a theoretical evaluation of MMW W-band (85–100 GHz) attenuation/scattering and power transfer in brownout conditions. The attenuation, reflectance, and backscattering of such media are important parameters in the design of active and passive W-band imaging and remote sensing.

The solution includes attenuation/scattering prediction and radiant flux, or power collected by the receiver. We are considering the case of sand grain clouds created by helicopter rotor airflow during landing in arid areas.

The predicted values from the mathematical model are compared with findings in the field and the literature [10,11,12,13,14,17]. The basic mathematical model for attenuation is the Mie theory for single spherical particle scattering. The propagation effects are modeled by volumetric integration of scattering by individual particles. The solution is carried out using the Rayleigh approximation. The method depends largely on the wave number, particulate radius, size distribution, and its dielectric constant.

In order to verify the validity of the proposed solution for the prediction of signal attenuation due to brownout, a comparison between attenuation predicted by different theoretical models with measured attenuation at 40 GHz (Ka-band) [14] is presented.

### 1.2. Objectives of the Current Work and Problem Statement

The current work aims at developing a simple model for assessing the MMW (85–100 GHz) attenuation/scattering and power transfer in brownout conditions.

The goal is to estimate the radiances of signals and noises that are incident on the entrance diameter of the receiving system. For that, the MMW extinction/scattering by the brownout should be evaluated. 

We are considering the case of brownout clouds created by helicopter rotor airflow during landing in arid areas. The evaluated scenarios are brownout environments over ranges up to 50 m. 

In the calculations, an active imaging system with a monochromatic source to produce the incident field is assumed. As the surface emissivity variation can be quite small in the mm-wave range, a passive imaging system may not be efficient enough. 

**Figure 1:**
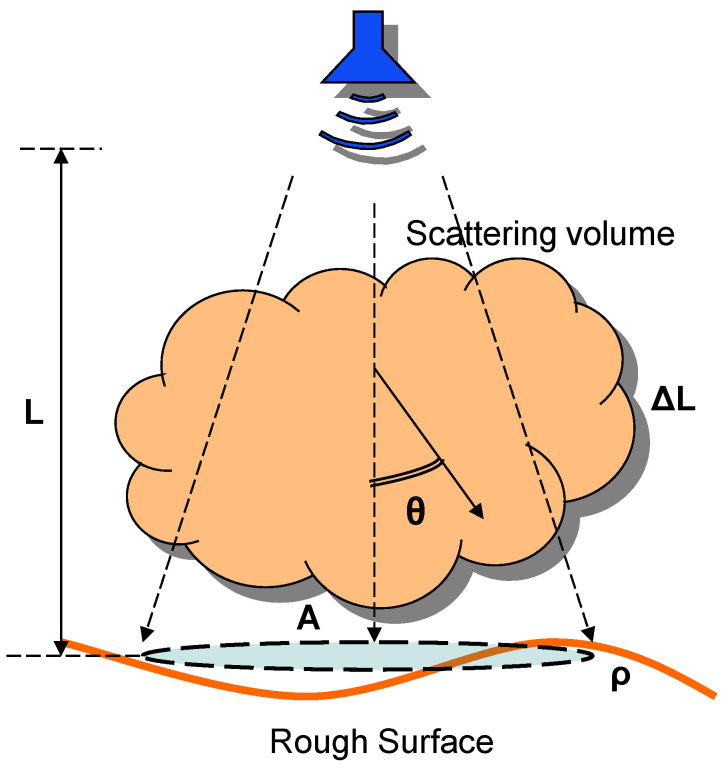
Schematic of brownout scattering problem and scene geometry.

Figure 1 shows the scene geometry. Calculations refer to the receiver aperture plane and not to the image plane.

It is assumed that the source emits a uniform radiance in a cone of half-aperture. It illuminates a scene area (*A*) at the given position in the surface plane located at range *L* from the mm-wave transceiver. The brownout is assumed to be separated by a small distance from the pupil plane. The thickness of the brownout is Δ*L*. The scene to be imaged/detected is located at a distance *L* from the imaging system. It is assumed that the imaged scene is Lambertian (nonspecular reflection), and its albedo is *ρ*.

The measured signal is the power backscattered by the scene to be imaged/detected. In addition, the source emission generates noise as it is backscattered by particulates of the brownout. The working frequencies were chosen within a transmission window of the MMW band between 85–100 GHz (W-band).

## 2. Materials and Methods

### 2.1. Attenuation Properties of Brownout at MMW

In general, the MMW attenuation or scattered intensity by sand/dust clouds at any angle is sensitive to the size distribution of cloud particles, their concentration, dielectric constant, and radiation wavelength.

#### 2.1.1. Attenuation Coefficient Estimation

The scattering/extinction coefficient for monodisperse particles is given by the relation

(1) $$\alpha_{ext,sca}=N\cdot C_{ext,sca}\left(\lambda ,\varepsilon ,r)\;[m^{-1}\right]$$

and the backscattering coefficient is

(2) $$\beta_{\pi }=N\cdot C_{sca}\left(\lambda ,\varepsilon ,r\right)\cdot \frac{P\left(\pi ,r\right)}{4\pi }\;[m^{-1}\;{\mathrm{sr}}^{-1}]$$

where *N* is the total concentration of particles [m^−3^], *C_ext,sca_*(*λ,ε,r*) [m^2^] is the effective extinction/scattering area or extinction/scattering cross-section of the particle of radius *r* with dielectric constant *ε*, *ε~m*^2^, where *m* is the refractive index. The ratio of extinction or scattering cross section to geometrical cross-section of particle *πr*^2^ is the efficiency factor for extinction/scattering, *Q_ext_ = C_ext/_πr*^2^.

The backscattering *β_π_* [m^−1^ sr^−1^] and the scattering coefficients *α_sca_* [m^−1^] are coupled via the backscattering phase function *P*(*π*,*r*), i.e., the phase function into 180° or π—direction, by the expression *β_π_ = α_sca_ P*(*π*,*r*)/4π.

In general, the angular scatter coefficient is given by

(3) $$\alpha_{S}\left(\theta \right)=\alpha_{sca}\cdot \frac{P\left(\theta \right)}{4\pi },$$

or

(4) $$\alpha_{S}\left(\theta \right)=\alpha_{ext}\cdot \omega_{0}\cdot \frac{P\left(\theta \right)}{4\pi },$$

where *P*(*θ*) [dimensionless] is the relative angular distribution of the radiant intensity scattered by a volume element, so-called scattering diagram, or phase function. It is normalized by $\frac{1}{4\pi }\int_{4\pi }P\left(\theta \right)\Omega d=1$.

*α_sca_* [m^−1^] is the volume total scattering coefficient $\alpha_{sca}=\int_{4\pi }\alpha \left(\theta \right)d\Omega$;

*α_ext_* is the particulate volume extinction or attenuation coefficient [m^−1^], ω_0_ = *α_sca_*/*α_ext_* is the single scattering albedo. The volume extinction coefficient includes the loss effects of both scattering and absorption.

The extinction/scattering [m^−1^] of MMW by particles of different sizes at a given wavelength can be expressed by the integral equation:

(5) $$\alpha_{ext,sca}=\int C_{ext,sca}\left(r,\lambda ,\varepsilon \right)\cdot N\left(r\right)dr,$$

or

(6) $$\alpha_{ext,sca}=\int \pi \cdot r^{2}\cdot Q_{ext,sca}\left(r,\lambda ,\varepsilon \right)\cdot N\left(r\right)dr,$$

and

(7) $$\beta_{\pi }=\int C_{sca}\left(r,\lambda ,\varepsilon \right)\cdot N\left(r\right)\cdot \frac{P\left(\pi ,r\right)}{4\pi }dr,$$

where *N*(*r*) is the particle size distribution per unit volume [m^−3^ μm^−1^] with radii between *r* and *r + dr*, *Q*(*r,λ,ε*) is the extinction efficiency and can be calculated by using the Mie theory. The single-scattering properties of particles are averaged over particle size distributions to obtain the bulk scattering properties of media (brownout, sandstorm, etc.).

The attenuation coefficient in [dB/km] or [dB/m] can be expressed as

(8) $$\alpha_{dB}=4.343\cdot \alpha_{ext}\cdot b,$$

where *α_ext_* [m^−1^] is the extinction calculated by (1) or (5); *b* is the constant, *b* = 1000 for *α_dB_* in [dB/km] and *b* = 1 for *α_dB_* in [dB/m].

Based on Rayleigh scattering approximation, Goldhirsh [10] developed a model for microwave attenuation in dust/sandstorms. The specific attenuation coefficient (dB/km) is expressed in terms of visibility by

(9) $$\alpha_{dB}=2.317\cdot 10^{-3}\cdot \frac{1}{V^{1.07}\lambda }\cdot \frac{\varepsilon^{\prime \prime }}{{\left(\varepsilon^{\prime }+2\right)}^{2}+\varepsilon^{\prime \prime 2}},$$

where *V* is the visibility in km, *λ* is the wavelength in meters, and *ε′* and *ε″* are the real and imaginary contributions of the relative dielectric constant of the dust/sand particles, respectively. In (9), the visibility is related to the relative mass of dust per m^3^ with applicable values for the region of Sudan [8].

The attenuation prediction model for dust storms may be expressed by different formulations as [10]

(10) $$\alpha_{dB}=2.457\cdot 10^{5}\cdot \frac{u_{r}}{\lambda }\cdot \frac{\varepsilon^{\prime \prime }}{{\left(\varepsilon^{\prime }+2\right)}^{2}+\varepsilon^{\prime \prime 2}},$$

where *λ* is the wavelength in meters and *u_r_* is the total relative volume of all dust particles per cubic meter of air expressed by

(11) $$u_{r}=\frac{4}{3}\pi \sum_{i}^{n}N_{i}\cdot r_{i}^{3},$$

where *N_i_* is the number of particles with different radii per m^3^ and *r_i_* is the particulate radius belonging to the *i*th bin of a particle size distribution. The expression (10) is applicable at wavelengths for which the Rayleigh condition is applicable.

Ali and Alhaider [11] derived an expression for the attenuation of radio waves propagating through airborne sand particles of permittivity *ε* and effective radius *r_e_* as

(12) $$\alpha_{dB}=0.19\cdot 10^{3}\cdot \frac{r_{e}}{V\cdot \lambda }\cdot \frac{3\varepsilon^{\prime \prime }}{{\left(\varepsilon^{\prime }+2\right)}^{2}+\varepsilon^{\prime \prime 2}},$$

where *r_e_* is the effective particle radius (the ratio of the third to second moments of the particle size distribution), *V* is the visibility in km, and λ is the wavelength measured in the same units as the particle size *r_e_.* Ahmed et al. [12] derived the same attenuation formula (12) for MMW propagation in sand and dust storms in an alternate way based on the measured probability density function and Mie scattering theory.

Based on the volumetric integration of the Mie scattering results by individual particles, Elabdin et al. [13,17] derived another attenuation prediction model for microwave propagation in dust storms. The attenuation coefficient (one way) is a function of the real and complex permittivities and the particle size distribution and is inversely proportional to the wavelength.

#### 2.1.2. Visibility and Total Particle Density

In order to estimate the attenuation and backscattering, data for the size distribution of particles are required, which is difficult to measure accurately. A way to characterize the density of brownout or sand/dust particulates can be according to the visibility of targets within the environment. This is still a fairly subjective measure as visibility is defined as the range at which a high contrast target can just be seen in the visible.

In the case that information on visibility is available, it can be related to the particulate density or concentration. The visibility *V* [km] can be expressed by the equation:

(13) $$V=\frac{\ln\left(1/B\right)}{\alpha_{0}},$$

where α_0_ is the optical extinction [km^−1^], and *B* is the threshold contrast defined as the percentage difference in the luminance between a target and reference background. Usually, *B* is chosen to be 0.02. In the experiments, a median value of *B* was found to be 0.031 [18]. We will use *B* = 0.031 in the calculations below.

The total particle density, *N_T_* [m^−3^], was expressed in terms of the visibility and the effective radius as [9]

(14) $$N_{T}=\frac{5.54\times 10^{-4}}{V\cdot r_{e}^{2}},$$

where *V* is in [km] and *r_e_* is the equivalent particle radius in [m].

The equivalent particle radius *r_e_* is defined as that radius for the monodisperse case, which gives the same attenuation as the polydisperse case assuming the given particle size distribution and *N_T_*. In the polydisperse case, using the particle size distribution function, the equivalent particle radius *r_e_* or effective radius is given by

(15) $$r_{e}=\frac{\int_{rmin}^{rmax}r^{3}N\left(r\right)dr}{\int_{rmin}^{rmax}r^{2}N\left(r\right)dr},$$

Assuming that particle size distribution in real brownout conditions may be replaced by an equivalent particle *r_e_*, the extinction/scattering characteristics can be estimated for a given wavelength and propagation scenario.

#### 2.1.3. Attenuation Cross-Section Model

The size range of brownout particulate radii is, in general, from ~0.5 μm to 300 μm. For the mm-wave band (W-band) of *f* = 80–100 GHz, the particle radius is smaller than the wavelength. Therefore, the Rayleigh approximation can be used for calculations of particulate extinction/scattering characteristics. For the proposed wavelengths or wave frequencies *f* ≤ 100 GHz, the expression for total extinction cross-section (*C_ext_*, [m^2^]) in the Rayleigh approximation for a spherical particle of radius *r* can be represented as [19]:

(16) Cext(r)=−4π⋅k⋅Im[ε−1ε+2]⋅r3+8π3⋅k4⋅Re[ε−1ε+2]2⋅r6,

where *r* is the particle radius [m]; *k* is the wave number (*k* = 2*π/λ*), *λ* is the wavelength [m] and *ε* is the dielectric constant of the particles, *ε = ε′ −* j*ε″*. Note that *ε* = *m*^2^, where *m* is the refractive index.

The Rayleigh equation for scattering cross-section, *C_sca_* [m^2^], is given by [19]

(17) $$C_{sca}\left(r\right)=\frac{8\pi }{3}\cdot k^{4}\cdot {\left|\frac{\varepsilon -1}{\varepsilon +2}\right|}^{2}\cdot r^{6},$$

The Rayleigh approximation provides a reasonable estimate for attenuation and scattering. Determining a more precise value requires one to perform the Mie scattering calculations. However, for mm-wave system design calculations, the Rayleigh approach is sufficient and much simpler to implement. The error between the Rayleigh approximation and the Mie calculations is <5% for the case of *k∙r* < 0.4.

The essential helicopter brownout occurred within arid conditions devoid of vegetation and is characterized mainly by sandy particles. The complex relative permittivities of sand and dust are adopted from the literature [5,13,15,20,21,22,23] at frequencies of 3–100 GHz.

Table 1 represents the dielectric constants for brownout particulates for semi-arid and desert areas used for calculations.

The real part of the relative dielectric constants of rocks varies within the range 3–8, with a weak dependency on frequency.

**Figure 2:**
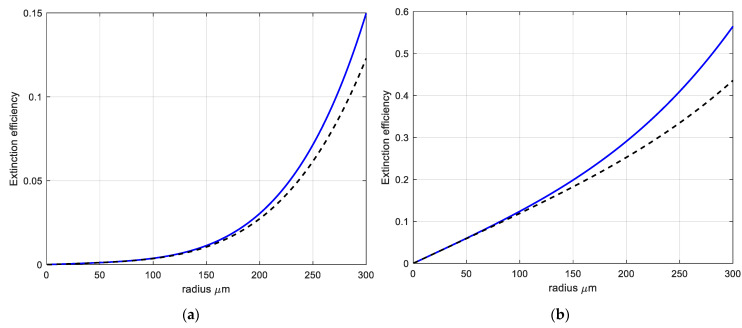
Extinction efficiency *Q_ext_ = C_ext/_πr*^2^ for 94 GHz and Brownout Desert particles with dielectric constant: (**a**) *ε* = 5.5 − j5.15 × 10^−2^; (**b**) *ε* = 3.5 − j1.65. The solid line (**―**)—Mie solution; dashed line (**— —**)—Rayleigh approximation (16).

Figure 2a,b shows examples of extinction efficiency calculated by (16) with different dielectric constants.

The Rayleigh approximation for MMW (W-band) extinction starts to diverge strongly from the Mie solution after about 170–200 μm particle radius, depending on the dielectric constant. The imaginary part of the dielectric constant is responsible for wave absorption. The extinction efficiency is about ten times different for the particle cloud, with two orders of magnitude difference in the imaginary part of permittivity (see Figure 2).

#### 2.1.4. Phase Function Model

For proposed wavelengths or wave frequencies *f* ≤ 100 GHz, the phase function can be approximated by a simple model, which is a modification of the Henyey-Greenstein approximation [24,25]:

(18) $$P\left(\mu ,g\right)=3\sqrt{\pi }\cdot \frac{1-g^{2}}{2+g^{2}}\cdot \frac{1+\mu^{2}}{{\left(1+g^{2}-2g\mu \right)}^{3/2}},$$

where $g=\frac{1-K}{\pi \sqrt{2}}$; *μ* = cos*θ* with 0 < *θ* < π [rad] is the scattering angle; *P*(*μ,g*) is normalized as $\int_{4\pi }P\left(\theta \right)\Omega d=4\pi$. *g* is the asymmetry factor with constant K, which can be modeled as follows (see Table 2):

K = b_1_ + b_2_∙X + b_3_∙X^2^ + b_4_∙X^3^, (19)

where X = 2π/*λ∙r*; valid for X < 0.7.

Thus, for a given wavelength, particulate radius, and dielectric constant, the angular distribution of scattered radiation can be estimated.

**Figure 3:**
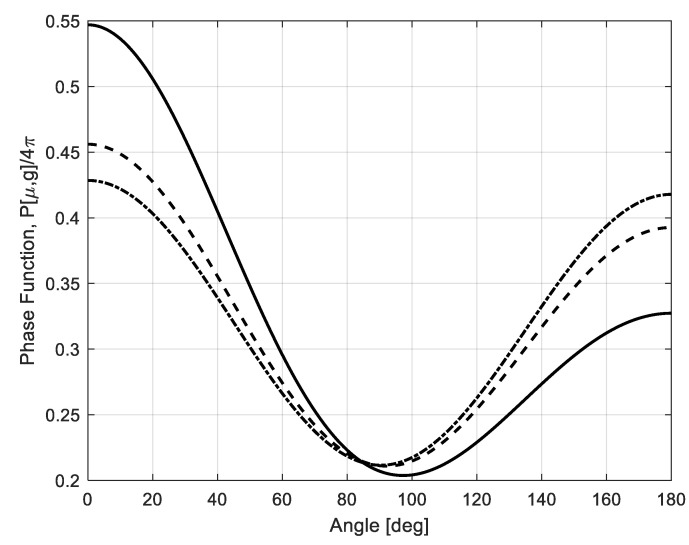
Phase function behavior for different particle radii: *r* = 60 μm (dash-dot **− ∙**), *r* = 150 μm (dashed line **— —**), *r* = 300 μm (solid line **―**); 94 GHz and brownout desert particles with dielectric constant *ε* = 5.5 − j5.15 × 10^−2^.

Figure 3 shows the phase function (18) behavior for different particle radii at 94 GHz and brownout desert particles with dielectric constant *ε* = 5.5 − j5.15 × 10^−2^.

The phase function calculations for W-band show that the Rayleigh approximation is not valid after about *r*~170 μm particle radius. The forward scattering starts to be more pronounced (see Figure 3).

### 2.2. Power Transfer in Brownout

#### 2.2.1. Signal Backscattered by the Scene

The power that is collected by the receiver is the portion of the scattered radiation from the surface that falls into the acceptance cone of the receiver defined by solid angle Ω*_R_*:

(20) $$I_{R}\left(L\right)=I\left(L\right)\cdot \rho /\pi \cdot \Omega_{R}\cdot T\left(L\right)$$

where Ω*_R_* = *A_R_*/*L*^2^ [sr] is the solid angle subtended by the receiver with the apex at an element of the surface; *I_R_* is the power at the receiver [W]; *A_R_* is the area of the receiver [m^2^]; *L* is the distance to the receiver from the surface [m]; *T*(*L*) is the atmospheric transmission from scene to the receiver; *ρ* is average surface reflectivity or albedo; *ρ/π* for Lambertian reflecting surface; *I(L)* is the irradiance or radiant flux density at a given position in the surface plane located at range *L* from the mm-wave source. It is given by

(21) $$I\left(L\right)=\frac{I_{0}\cdot T\left(L\right)}{A\left(L\right)},\;[W\;m^{-2}]$$

where *I*_0_ is the total power of the MMW source [W]; *A*(*L*) is the scene area [m^2^] illuminated by the MMW source at distance *L*.

The atmospheric transmittance *T* follows the exponential law of attenuation, the Beer-Lambert law. It is related to the extinction (1) and (5) by

(22) $$T\left(L\right)=e^{-\alpha_{ext}L},$$

Thus, the signal radiance backscattered by the scene at the receiver is given by

(23) $$I_{R}\left(L\right)=\frac{I_{0}}{A(L)}\cdot \rho /\pi \cdot \frac{A_{R}}{L^{2}}\cdot T^{2}\left(L\right)$$

In the general case, the actual backscatter from a surface depends on a number of terrain parameters. The surface roughness and moisture content that may influence surface reflectivity *ρ* or albedo must be taken into account. For imaging systems, portions of image plane irradiance from the illuminated surface should be calculated for given system parameters (field-of-view, detector & pixel size, etc.).

#### 2.2.2. Noise Backscattered by the Brownout

The power removed from the primarily transmitted radiation due to scattering reappears as a radiation source (scattering volume in Figure 1) and, thus, as background at the receiver.

The radiant intensity scattered by unit volume with thickness Δ*L* per unit solid angle in the direction of *θ* (centered around *θ*) relative to the direction of the incident beam is given by [26]

(24) $$I\left(\theta \right)=I_{0}\cdot \Delta L\cdot \alpha_{S}\left(\theta \right)\cdot T,\;[W\;{\mathrm{sr}}^{-1}]$$

The radiant flux that is collected by the receiver is the portion of the scattered radiation from the cloud of particulates that falls into the acceptance cone of the receiver defined by solid angle Ω*_R_*:

(25) $$I_{B}=I\left(\theta \right)\cdot \Omega_{R}\cdot T,$$

or

(26) $$I_{B}=I_{0}\cdot \Delta L\cdot \alpha_{S}\left(\theta \right)\cdot \frac{A_{R}}{L^{2}}\cdot T^{2},$$

where Δ*L* is the length of the scattering volume [m]; *θ* is the scattering angle, 0 < *θ* < π [rad]; *I*(*θ*) is the intensity of angularly scattered radiation (radiant intensity) [W sr^−1^]; α_S_(*θ*) is the directional scattering coefficient [m^−1^ sr^−1^]. In the case of backscatter: *α_S_*(*θ*) ≡ *α_S_*(π) ≡ *β_π_* is the backscatter coefficient.

Thus, the radiance of noise backscattered by brownout into the receiver aperture is represented by [26]

(27) $$I_{B}=I_{0}\cdot \Delta L\cdot \beta_{\pi }\cdot \frac{A_{R}}{L^{2}}\cdot \exp\left(-2\alpha_{ext}L\right),$$

#### 2.2.3. Brownout Particle Size Distribution Model

The particulate size distribution (PSD) in brownout conditions for different types of helicopters is given in [15,21,22,23]. In the brownout PSD model [21], the particulate size distribution can be represented by the sum of linear and log-normal distributions:

(28a) $$n_{1}\left(r\right)=N_{1}\cdot \frac{{\left(r\right)}^{-3.236}}{\int_{r1}^{rn}{\left(r\right)}^{-3.236}dr},$$

(28b) $$n_{2}\left(r\right)=N_{2}\cdot \frac{1}{\ln\left(10\right)\cdot r\cdot \sigma \cdot \sqrt{2\pi }}\cdot \exp\left\{-\frac{{\left[\log\left(r\right)-\log\left(r_{m}\right)\right]}^{2}}{2\sigma^{2}}\right\},$$

where *n(r)* is the cumulative number density of particles of radius *r*, *σ* is the standard deviation, *r_m_* is the mode radius [μm], and *N*_1,2_ is the number density of particles. The range of particle radii is chosen to be *r*_1_ = 0.5 μm and *r_n_* = 300 μm.

The parameters of the size distribution given by (28a), (28b) are presented in Table 3.

The choices of *N_i_* in Table 3 are normalized to correspond to 1 particle/m^3^, i.e.,

(29) $$\int_{r1}^{rn}\left[n_{1}\left(r\right)+n_{2}\left(r\right)\right]dr=1$$

Total particle concentration is *N_total_* = 5 × 10^9^ m^−3^. It was estimated from [6,8]. The model is represented by

(30) $$N\left(r\right)=N_{total}\cdot \left[n_{1}\left(r\right)+n_{2}\left(r\right)\right],\;[m^{-3}{\mu m}^{-1}]$$

**Figure 4:**
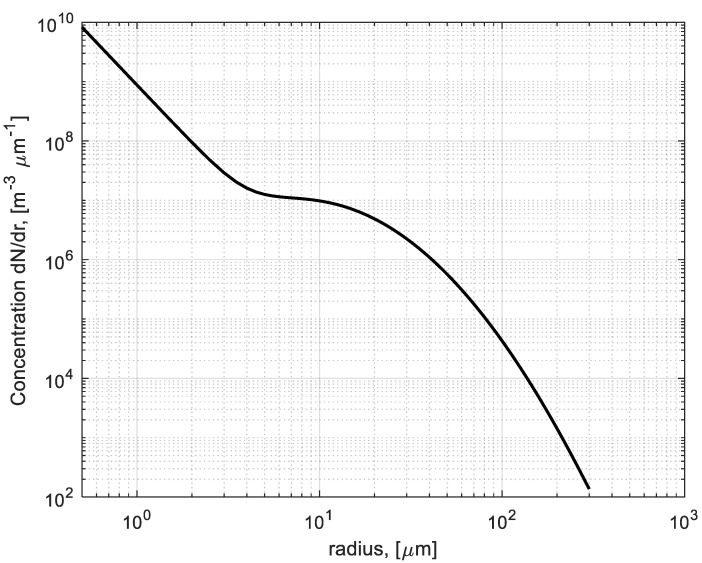
Particulate number distribution (m^−3^ μm^−1^) for the brownout model adopted from [21]. Total particle concentration fixed at *N_total_* = 5 × 10^9^ m^−3^.

The brownout particulate size distribution is shown in Figure 4. The model corresponds to the visibility in brownout of about *V* ~ 4 m for a particle range of *r* = 0.5–300 μm.

The equivalent particle radius for the brownout PSD model (30) is about *r_e_* ~ 58 μm.

## 3. Results

### 3.1. Comparison with Models and Measured Data

In order to verify the validity of the proposed solution for the prediction of signal attenuation due to brownout, a comparison run with the models developed by different investigators for the prediction of signal attenuation due to dust/sandstorms was performed. The results were also compared with published field measurements [14].

Calculated and measured attenuation values for visibility of *V* = 0.625 km at 40 GHz are given in Table 4. Measured attenuation [14] is compared for the same conditions to the predicted attenuation using the models by Goldhirsh [10], Equation (9), Elabdin et al. [13], Ali et al. [11], Equation (12), and proposed solution by Equations (1), (8), (14) and (16). The values considered in calculations are given in Table 4.

Unlike model (9), all models show satisfying agreement with the measured attenuation (Table 4).

**Figure 5:**
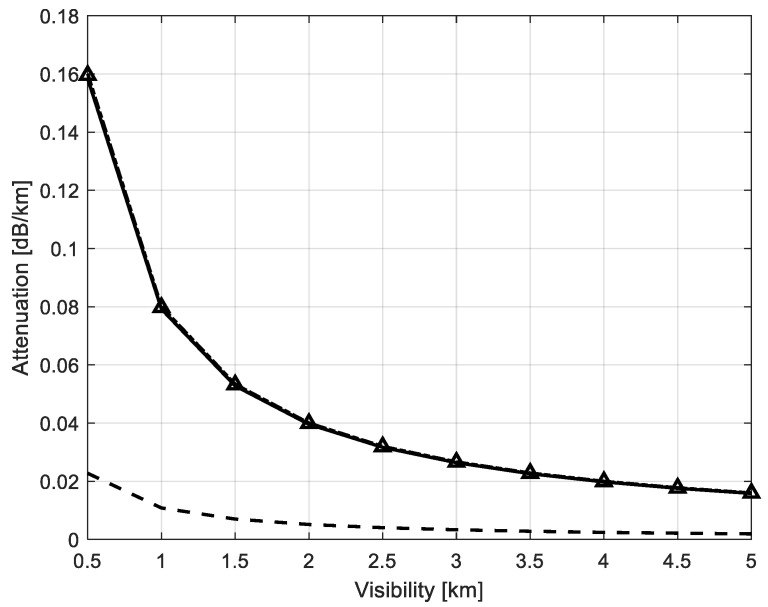
Comparison of specific attenuation due to dust storm for different values of visibility at 40 GHz; Goldhirsh [10] Equation (9)—dashed line (**— —**), Elabdin et al. [13]—solid line (**―**), Ali et al. [11] Equation (12)—triangles (**∆**), proposed solution by Equations (1), (8), (14) and (16).

The attenuation calculations versus different values of visibility plot at 40 GHz (Ka-band) for various models are shown in Figure 5. Unlike the predicted values by Equation (9), the attenuation values calculated by the proposed solution and different models show full agreement.

### 3.2. Power Transfer Estimation in Brownout

We examine here the MMW attenuation characteristics resulting from the passage of a W-band radar signal through a uniform dust/sand cloud region as obtained by different models (see Section 2.1.1) and using (23) and (27) for signal radiance at the receiver.

We shall use the proposed permittivity values and brownout particle size distribution as described previously (see Table 3 and Section 2.2.3). The attenuation results for different complex permittivity in dB/km for the proposed solution and models are given in Table 5.

The specific parameter values considered are 94 GHz transceiver frequency; visibility *V* = 0.004 km. Brownout PSD model (30), the equivalent particle radius *r_e_* = 58 μm.

The Goldhirsh model (9) shows values an order of magnitude smaller consistently for all data. The model (9) can be corrected by factor 10.

As mentioned in Section 2.1.1, in the Goldhirsh model (9), the visibility is related to the given mass concentration of dust in the region of Sudan. For other particulate concentrations, visibility, and dielectric constants, the Goldhirsh model (9) shows values an order of magnitude smaller consistently for all data (see Table 5). Thus, model (9) can be corrected by factor 10 and applied to different conditions.

Now we examine the echo power, *I_r_* and *I_b_,* given by (23) and (27). As a typical example for an existing MMW radar system, we assume the parameters given in [27]. Table 6 represents detailed specifications of the MMW link and transceiver parameters. It is assumed that the scene area [m^2^] illuminated by the MMW source at distance *L* has the same size as the receiver viewing area.

The extinction/scattering cross-sections and phase functions are estimated by (16), (17), and (18). Then the extinction and backscatter coefficients are calculated by use of (5) and (7). The power at the receiver backscattered from the surface and brownout cloud is calculated by use of (23) and (27). The calculations are compared to results obtained from Mie’s theory.

**Figure 6:**
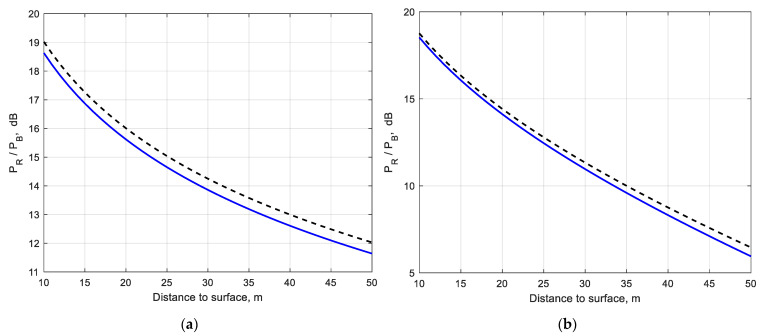
Scene-to-brownout backscatter ratio vs. distance to surface; solid line (**―**)—(23), (27) with Mie solution; dashed line (**— —**)—(23), (27) with Rayleigh approximation. Surface reflectance: *ρ* = 0.15; (**a**) *ε* = 5.5 − j5.15 × 10^−2^, (**b**) *ε* = 3.5 − j1.65.

Figure 6a,b shows the variation of the scene-to-brownout backscatter ratio (in dB) for different distances to the surface for visibility V = 4 m and brownout PSD (30). It is clear that the scene-to-brownout backscatter ratio increases with decreasing distance to the surface. For absorbing particles with increasing imaginary components of permittivity, the scene-to-brownout backscatter ratio decreases faster with increasing distance (Figure 6b). For example (see Figure 6a,b), the scene-to-brownout backscatter ratio at 50 m distance is Pr/Pb ~ 12 dB for particles with *ε* = 5.5 − j5.15 × 10^−2^. It is reduced to 5 dB for particles with *ε* = 3.5 − j1.65.

**Figure 7:**
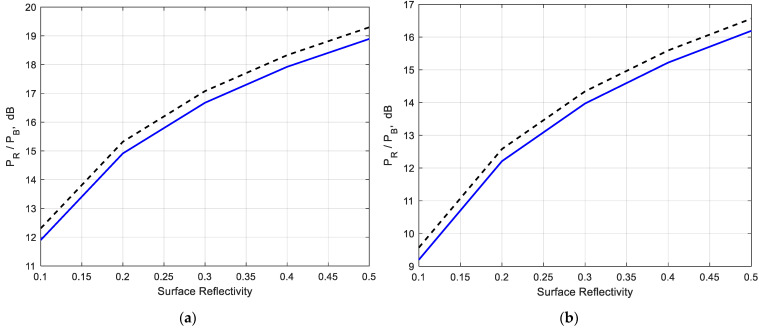
Scene-to-brownout backscatter ratio vs. surface reflectance; solid line (**―**)—(23), (27) with Mie solution; dashed line (**— —**)—(23), (27) with Rayleigh approximation. Distance to the surface *L* = 30 m; (**a**) *ε* = 5.5 − j5.15 × 10^−2^; (**b**) *ε* = 3.5 − j1.65.

The variation of the scene-to-brownout backscatter ratio with surface reflectance at a given distance (30 m) from the transceiver is shown in Figure 7a,b. Surface reflectance increasing leads to a growth of the scene-to-brownout backscatter ratio.

**Figure 8:**
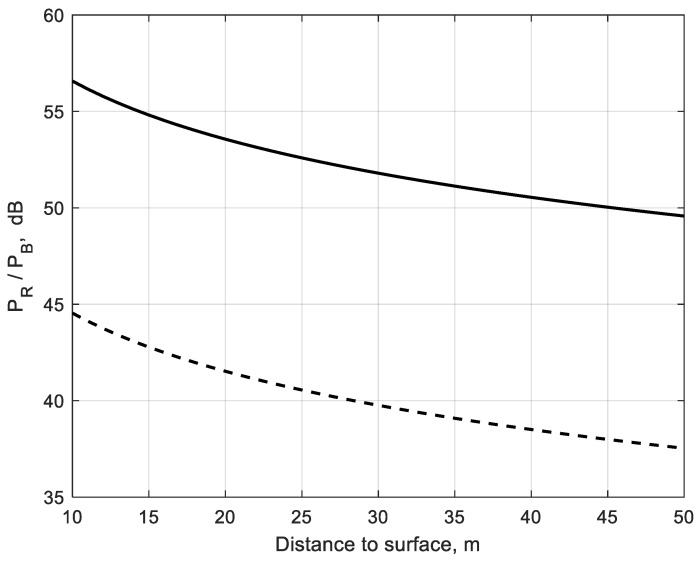
Scene-to-brownout backscatter ratio vs. distance to the surface for different equivalent brownout particles: *r_e_* = 15 μm (solid line **―**); *r_e_* = 30 μm (dashed line **— —**). Surface reflectance *ρ* = 0.15; *ε* = 5.5 − j5.15 × 10^−2^; Visibility = 10 m.

Particulate scattering/absorption properties presented by complex permittivity influence the signal attenuation/scattering at W-band. Assuming that particle size distribution in real brownout conditions may be replaced by an equivalent particle *r_e_*, the extinction/scattering characteristics can be estimated for a given wavelength and propagation scenario. Figure 8 shows the results of scene-to-brownout backscatter ratio calculation vs. distance to surface assuming different equivalent brownout particles (*r_e_* = 15 μm and *r_e_* = 30 μm) and visibility = 10 m.

As shown in Figure 8, the scene-to-brownout backscatter ratio may vary significantly as a result of various equivalent particulate sizes for the same visibility. It may correspond to different scenarios of a helicopter landing in brownout conditions.

## 4. Conclusions

In the present work, we proposed a simple model for MMW (85–100 GHz) attenuation/scattering and power transfer estimation in brownout conditions. In addition, we proposed a simple model for the phase function calculation, which is a modification of the Henyey-Greenstein approximation for 2π/λ∙*r* < 1 and for different dielectric constants.

It was shown that the Rayleigh approximation for MMW (W-band) extinction starts to diverge strongly from the Mie solution after about 170–200 μm particle radius, depending on the dielectric constant.

The phase function calculations for W-band show that the Rayleigh approximation is not valid after about *r* ~ 200 μm particle radius. The forward scattering starts to be more pronounced (see Figure 2). Thus, for sub-THz wave bands above ~100 GHz and particulate radii above ~200 μm, the Rayleigh approximation is not valid.

In order to verify the validity of the proposed solution for the prediction of signal attenuation due to brownout, a comparison run with the models developed by different investigators for the prediction of signal attenuation due to dust/sandstorms were performed. The results were also compared with published field measurements [14] and showed satisfactory agreement.

The proposed model for MMW attenuation and power transfer estimation can be used for investigations and simulations. The main contributions to the radiance backscattered by scene and by brownout are determined analytically. The proposed solution gives a faster alternative for power balance estimation.

## Figures and Tables

**Table 1:** The dielectric constants for brownout particulates.

Particulate Type	Dielectric Constant *ε* = *ε*′ − j*ε*″	Refractive Index m = √*ε*
Brownout Desert [21,22,23]	5.5 − j5.15 × 10^−2^	2.34 − j0.011
Brownout Mid-Latitude [21,22,23]	3.5 − j4.83 × 10^−2^	1.86 − j0.013
Sand & dust-storm [13]	3.5 − j1.64	1.92 − j0.43
Sand & Dust [15]	3.0 − j0.6	1.74 − j0.17
Sand (Libya) [20]	4.96 − j0.135	2.23 − j0.303

**Table 2:** Asymmetry factor constant K coefficients.

Constant	Real (*ε*) ≥ 4.5	2.0 < Real (*ε*) < 4.5
b1	0.9996	0.9996
b2	1.82 × 10^−2^	1.465 × 10^−2^
b3	−1.54	−1.174
b4	0.7161	0.4865

**Table 3:** Particulate size distribution parameters.

*N_i_*	*r_m_* (μm)	*σ*	Type
0.96	--	--	Linear
0.04	14.6	0.33	Log-normal

**Table 4:** Result of measurements and calculations of attenuation [dB/km] due to dust storm; Frequency 40 GHz (Ka-band); Equivalent dust particle radius *r*_e_ = 30 μm.

Visibility, km	Dielectric Constant	Ref. [13]	Ref. [10]	Attenuation Ref. [11]	dB/km Proposed Solution *	Measured Ref. [14]
0.626	*ε* = 4 − j1.325	0.132	0.018	0.133	0.133	0.14

* Proposed solution—Equations (1), (8), (14), and (16).

**Table 5:** Attenuation dB/km models comparison for different complex permittivity.

Complex Permittivity *ε* = *ε*′ − j*ε*″	Ref. [13]	Equation (9)	Attenuation in Equation (10)	dB/km Equation (12)	*r_e_* = 58 μm *	PSD Equation (30) **
3.5 − j1.65	131.4	13.3	130.5	130.5	128	132.8
4.96 − j0.135	7.38	0.742	7.25	7.25	7.37	10.5
5.5 − j5.15 × 10^−2^	2.5	0.25	2.4	2.4	2.66	5.9

* Proposed solution—Equations (1), (8), (14), and (16), ** by using Equations (5), (8), and (16).

**Table 6:** Parameters for calculations.

MMW Link/Transceiver	Parameters
Distance to surface	*L* = 10 ÷ 50 m
Surface reflectance	*ρ* = 0.15
Receiver aperture diameter	*D*_R_ = 0.5 m
Transmitter power	*I*_0_ = 1 W
Frequency	*f* = 94 GHz
Brownout Desert model	*ε* = 5.5 − j5.15 × 10^−2^
Sand & dust-storm	*ε* = 3.5 − j1.65
Visibility [brownout PSD (30)]	V = 0.004 km
